# Inhibition of DNMT-1 alleviates ferroptosis through NCOA4 mediated ferritinophagy during diabetes myocardial ischemia/reperfusion injury

**DOI:** 10.1038/s41420-021-00656-0

**Published:** 2021-09-29

**Authors:** Wenyuan Li, Wei Li, Yao Wang, Yan Leng, Zhongyuan Xia

**Affiliations:** 1 Department of Anesthesiology, Renmin Hospital of Wuhan University, Wuhan, China; 2 Department of Infectious Diseases, Renmin Hospital of Wuhan University, Wuhan, China

**Keywords:** Myocardial infarction, Ion channel signalling

## Abstract

The purpose of this study was to investigate whether inhibition of DNA (cytosine-5)-methyltransferase 1 (DNMT-1) alleviated ferroptosis through nuclear receptor coactivator 4 (NCOA4)-mediated ferritinophagy during diabetes myocardial (DM) ischemia/reperfusion (I/R) injury (IRI). Rat DM + sham (DS), I/R, and DM + I/R (DIR), H9c2 cell high glucose (HG), hypoxia reoxygenation (H/R), and high-glucose hypoxia reoxygenation (HH/R) models were established. DNMT-1 inhibitor 5-Aza-2’-deoxycytidine (5-aza-CdR) was administered to rat and cell models. The protein level of DNMT-1, NCOA4, FTH, GPX4, Beclin-1, and P62 was detected by western blotting. Compared with normal sham (NS) group, myocardial tissue was injured in DS and I/R models. The level of DNMT-1, NCOA4, and ferroptosis was increased. Moreover, the cell injury was more serious in rat DIR or HH/R model. 5-Aza-CdR could reduce NCOA4-mediated ferritinophagy and myocardial injury in DIR and HH/R models. Moreover, the siRNA for NCOA4 could also reduce the level of ferritinophagy and cell injury in HH/R model. 5-Aza-CdR enhanced the protective effect for NCOA4-siRNA in the process of cell injury. Inhibition of DNMT-1 could reduce ferroptosis during DIR, which the NCOA4-mediated ferritinophagy might be regulated.

## Introduction

Diabetes is closely related to cardiovascular disease and is an independent risk factor for cardiovascular complications and mortality after myocardial infarction [1]. Diabetes increases the risk of cardiovascular disease, which is the main reason for the increased incidence of coronary artery disease and stroke, and increased risk of heart failure. Ischemic heart disease is the leading cause of heart failure and death in patients with heart disease worldwide. Rapid restoration of blood supply to ischemic tissue is the standard clinical treatment for myocardial infarction. However, this may lead to oxidative damage to cells and additional cell death, called “ischemia-reperfusion (I/R) injury (IRI)” ([2]). At present, reducing reperfusion injury after myocardial infarction is a hotspot in clinical research for the treatment of myocardial IRI.

DNA methylation is a major epigenetic modification, which is under the catalysis of DNA methyltransferase (DNMT), and *S*-adenosylmethionine was used as the methyl donor. Then the activated methyl is added to the 5’-carbon end of cytosine. It is modified to 5-methylcytosine (5mC) [3]. 5mC is mostly located in the cytosine phosphate guanine island of the promoter. Hypermethylation in this region usually leads to downregulation of gene expression. DNA methylation can cause changes in chromatin structure, DNA conformation and stability, and the way DNA interacts with proteins, thereby controlling gene expression [3]. A large number of studies have shown that DNA methylation is closely related to the occurrence and development of cardiovascular and cerebrovascular diseases, diabetes, tumors, and other diseases [4–6]. However, the relationship between DNMT-1 and diabetic myocardial IRI has not been reported.

Autophagy is a process in which organelles and proteins in cells are decomposed by lysosomes under the stimulation of a series of factors such as starvation, nutritional deficiencies, growth factor deficiencies, energy deficiency, or stress death threats. Autophagy that exists in cells is conducive to cell metabolism and renewal, and can maintain cell survival, differentiation, development, and homeostasis [7]. Ferritinophagy, as a type of autophagy, has a similar physiological effect. Ferritinophagy was first proposed by Mancias et al. [8] and the researchers identified nuclear receptor coactivator 4 (NCOA4) as a selective autophagy by quantitative proteomics. The receptor of phagocytosis, which mediates the degradation of ferritin in the autophagosome, causes the release of iron ions bound to ferritin, to become free iron, and this process is called ferritinophagy. The studies have shown that iron derived from autophagy-mediated ferritin degradation induces cardiomyocyte death and heart failure in mice [9]. Further, ferritinophagy is involved in apelin-13-induced cardiomyocytes hypertrophy [10]. However, the relationship between ferritinophagy and diabetic myocardial IRI has not been reported. Whether DNMT-1 can affect ferroptosis through NCOA4 is worthy of further study in the process of diabetes myocardial IRI.

### Cell model and drug administration

H9c2 cells were cultured in Dulbecco’s modified Eagle’s (DMEM) low-sugar medium containing 10% fetal bovine serum (FBS) under the cell culture chamber, containing 10% CO_2_ at 37 °C. When H9c2 cells grew to 70–80% density, the cells were digested with trypsin. Then the cells were planted in six-well plates. H9c2 cells were divided into normal group, high-glucose (HG) group, hypoxia reoxygenation group (H/R), HG H/R group (HH/R), and HH/R + 5-Aza-2’-deoxycytidine (5-aza-CdR) group. After being synchronized with serum-free low-sugar DMEM for 24 h, HG medium (glycol concentration of 30 mmol/L) was added. Then, the cells were cultured at 37 °C for 24 h to establish an HG model. The H/R model was established by hypoxia (volume fraction 94% N_2_ + volume fraction 5% CO_2_ + volume fraction 1% O_2_) 4 h and reoxygenation (volume fraction 90% atmosphere + volume fraction 10% CO_2_) 2 h. In the last 6 h for building the HG model, the cells were placed to hypoxia 4 h and reoxygenation 2 h to establish a HH/R cell model. 5-Aza-CdR affected at a concentration of 30 μmol/L for 24 h before building cell models [11].

### siRNA transfection in H9c2 Cells

NCOA4 small interfering (siRNA) and control-siRNA oligonucleotides for rat species were synthesized by Guangzhou RiboBio Co., Ltd, Guangzhou, China (Table 1). The DNMT1-siRNA was purchased from Thermo Fisher Scientific (Waltham, MA, USA). H9c2 cells were passed into new plates to transfect with siRNA through using Lipofectamine 2000. After 6 h, the medium was changed and the cells grew for another 48 h. The effect siRNA for the knockdown of NCOA4 was confirmed by western blotting.

**Table 1:** siRNA sequence of NCOA4.

siRNA	Sequence	
NCOA4-1	Sense	5′-GCUGUUUCUCUCAGUCAAUTT-3′
	Anti-sense	5′-AUUCACUGAGAGAAACAGCTT-3′
NCOA4-2	Sense	5′-GCCCUACAAUGUCAAUGAUTT-3′
	Anti-sense	5′-AUCAUUCACAUUGUAGGGCTT-3′
NCOA4-3	Sense	5′-CCAUCAGGACACAUGUAAATT-3′
	Anti-sense	5′-UUUACAUGUGUCCUGAUGGTT-3′
Negative control-siRNA	Sense	5′-UUCUCCGAACGUGUCACGUTT-3′
	Anti-sense	5′-ACGUGACACGUUCGGAGAATT-3′

### Animal model establishment and drug administration

Fifty specific pathogen-free male Sprague–Dawley rats (weighing 210–240 g) were purchased from Beijing Huakang Biotechnology Co., Ltd (Beijing, China). The experiment was conducted at a temperature of 25 °C ± 2 °C, relative humidity of 50% ± 15%, and normal circadian rhythm (12 h dark/12 h light) in Renmin Hospital of Wuhan University. All the rats got free water and food. The study protocols were in accordance with the internationally accepted principles and Guidelines for the Care and Use of Laboratory Animals of Wuhan University (Institutional Animal Care and Use Committee Issue number WDRM (welfare) 20180214). All the rats were randomly equally divided into five groups: normal + sham group (NS), I/R group (I/R), diabetes mellitus (DM) + sham (DS) group, DM + I/R group (DIR), and 5-aza-CdR group. Every group included ten rats. The rat models were built as follows.

The DS model was established by injecting 1% streptozotocin into the tail vein at 60 mg/kg dose. After 3 days, if the fasting blood glucose level was higher than 16.7 mmol/L, the DS model was successfully built. The NS and the I/R group were given 0.9% sodium chloride injection. Thereafter, the general conditions for normal and DM rats are showed in Table 2. After 8 weeks, all the rats were intraperitoneally injected with 1.5% sodium pentobarbital at a dose of 0.005 mL/g. They were given electrocardiogram (ECG) monitoring management. The rats in each group were intubated, connected to a ventilator, and mechanically ventilated at a frequency of 80–90 times/min.

**Table 2:** General condition of normal and DM rats.

Group	Body weight (g)	Food intake g/(kg/d)	Water intake ml/(kg/d)	Blood glucose (mmol/L)
Normal	395.0 ± 29.7	63.9 ± 6.2	109.4 ± 9.7	6.0 ± 0.5
DM	202.6 ± 17.5^*^	108.9 ± 9.8^*^	215.6 ± 16.3^*^	24.7 ± 3.7^*^

Data are presented as the means ± SD.

*DM* diabetic mellitus.

*P < 0.01, compared with normal rats.

The heart rate (HR) and blood pressure were recorded. The rats’ hearts were exposed by sternotomy along the left edge of sternum. The left anterior descending (LAD) coronary artery between the left atrial appendage and the pulmonary artery cone was sutured and then covered with saline gauze. If the left ventricle apex for myocardial blanched and ST segment of ECG was elevated, the LAD coronary artery was successfully ligatured. After 30 min of ischemia, the ligature was cut to restore the reperfusion of coronary arteries. At the same time, the anterior wall of the left ventricle turning red and the descending ST segment of ECG indicated the successful reperfusion. Then, the coronary arteries restored the reperfusion for 2 h. Rats in the NS group were only threaded without ligation. Referred to the reported dosage [12], DIR + 5-aza-CdR group rats were intraperitoneally injected with 5-aza-CdR at a dose of 0.25 mg/kg until 2 h before building I/R model. The part of the cardiac apex and serum was collected for the follow-up experiments.

### TTC determination of myocardial infarction area

The measuring of infarction/area at risk (AAR) followed the previously published steps [13]. At the end of reperfusion, six rats were randomly selected from each group, LAD was ligated again, and 3% Evans Blue 1 ml was injected into the left ventricular cavity. The residual blood was washed with pre-cooled saline and the dye was distributed in the myocardium through the coronary artery. The heart was quickly removed and stored at −20 °C for 2 h. The sections were cut perpendicular to the long axis of the myocardium and the thickness was 2 mm. The cells were incubated in 1% triphenyltetrazolium chloride (TTC) buffer at 37 °C for 15 min in the dark and then placed in 4% paraformaldehyde for 30 min. The blue staining part of Evans Blue is a viable myocardial tissue called normal myocardial tissue (non-ischemic myocardium), the TTC staining area is brick red (ischemic myocardium), and the non-TTC staining area is gray (infarcted myocardium). Each layer of the slice was photographed on both sides and the photos were analyzed by ImagePro Plus 6.0 software. The area of myocardial ischemic area and percentage of infarct area were measured as representative of the percentage of ischemic myocardium area to the left ventricular area (AAR/LV%) and the percentage of infarcted myocardium area to the area of ischemic myocardium (IA/AAR%).

### Cell viability detection

The cell suspension was planted evenly in the 96-well plate at a density of 5 × 10^3^ cells/100 μL per well. After the cells were being attached, they were modeled and administered with the corresponding drugs. The control group was set with only adding DMEM. Each group had six duplicate holes. Cell counting kit-8 (CCK-8) was added to the basal medium at a ratio of 1 : 10 to prepare CCK-8 working solution. Then, the culture solution was removed. CCK-8 working solution for 100 μL was added to each well. The cells were incubated for 1 h in the dark. At last, the optical density value for each well at 490 nm absorbance was measured by a microplate reader.

### CK-MB, ROS, LDH, GSH, and intracellular ferrous ion (Fe^2+^) determination

The levels of creatine kinase (CK)-MB, lactate dehydrogenase (LDH), glutathione (GSH), and intracellular Fe^2+^ in the cells and cell supernatant were measured according to the assay kits instructions. The intensity of each index was measured by a microplate reader. Following the instructions of reactive oxygen species (ROS) assay kit, the cells were incubated with 2′,7′-dichlorodihydrofluorescein diacetate probes for 30 min in the dark. The fluorescence microplate was used to observe fluoresce intensity. The stimulated light wavelength was 485 nm and the emission light wavelength was 525 nm. ROS level (%) = fluorescence value of intervention group/control group × 100% [14].

### Western blotting to detect protein expression

After the end of the intervention, total cell proteins and myocardial tissue were extracted. Bicinchoninic acid method was used to determine the protein concentration and quantify the total amount of protein. SDS-polyacrylamide gel electrophoresis was performed to separate the protein extracts. After the electrophoresis, the gel was transferred to polyvinylidene fluoride membrane. The membranes were incubated with 5% skim milk for 1 h. The primary antibodies (DNMT-1, 1 : 1000; NCOA4, 1 : 1000; P62, 1 : 1000; Beclin-1, 1 : 1000; LC3 1 : 1000; ferritin heavy chain (FTH), 1 : 1000; GSH peroxidase 4 (GPX4), 1 : 1000; glyceraldehyde 3-phosphate dehydrogenase (GAPDH), 1 : 2000) were added to incubate with the membranes at 4 °C overnight. Fluorescent secondary antibody was incubated for 1 h at room temperature. The blottings were at last analyzed with Odessay software (Li-Cor, Lincoln, NE, USA).

### Hematoxylin–eosin staining, TUNEL staining, and immunohistochemistry to detect myocardial tissue

The fresh myocardial tissues in each group were fixed with 4% paraformaldehyde. After being embedded with paraffin, the paraffin blocks were cut into 3 mm slices. Then, hematoxylin–eosin staining was used for assessing the morphological changes and damage degree. The protein expression level of NCOA4 in the myocardial tissue slices was detected by immunohistochemistry. The apoptotic rate of cells in myocardial tissue was evaluated by TdT- mediated biotinylated nick end labeling (TUNEL) Assay Kit (Roche Applied Science) according to the manufacturer’s instructions and then analyzed under a fluorescence microscope. There were six specimens of rats used to count the LI. The ×200 high magnification was counted in the fields. The conventional labeling index (LI) method was used. LI = number of positive cells in each visual field/all cells in the visual field. Three visual fields were selected. All cells in each visual field (including crypt and mucosa cells) were counted and the marker index of each visual field was counted. The apoptosis index of each group was equal to the average of each visual field marker index [15, 16].

### Immunofluorescence staining to detect protein expression

The cells were fixed with 4% paraformaldehyde. After being blocked with normal goat serum, the rabbit anti-rat NCOA4 (1 : 200) and GPX4 (1 : 200) antibodies were used for incubating the cells overnight at 4 °C. Fluorescein isothiocyanate (FITC)- and Cy3-labeled goat anti-rabbit secondary antibodies (1 : 50) were incubated for 1 h at 37 °C in the dark. 4’,6-Diamidino-2-phenylindole was added to incubate for 5 min. The magnification light microscope (Olympus, Tokyo, Japan) was used to take images at ×200 fields. The fluorescence intensity was analyzed with Image J software.

### Statistical analysis

The data and statistical analysis comply with the recommendations on experimental design and analysis in pharmacology. All data were analyzed blindly using SPSS 17.0 software and data are shown as mean ± SEM. For the ROS production, data were normalized to the mean values of the control group to reduce unwanted sources of variation. The group size for data subjected to statistical analysis was *n* ≥ 6, where *n* is the number of mice in each group or the number of separate experiments (in vitro), and statistical analysis was done using these biological replicates. The comparison between two groups was analyzed with Student’s *t*-test. The comparison among three or more groups was performed by one-way analysis of variance (with post hoc analysis by Bonferroni correction). *P* < 0.05 indicated the statistical difference.

## Results

### DS, I/R, and DIR rat model construction and verification

**Fig. 1:**
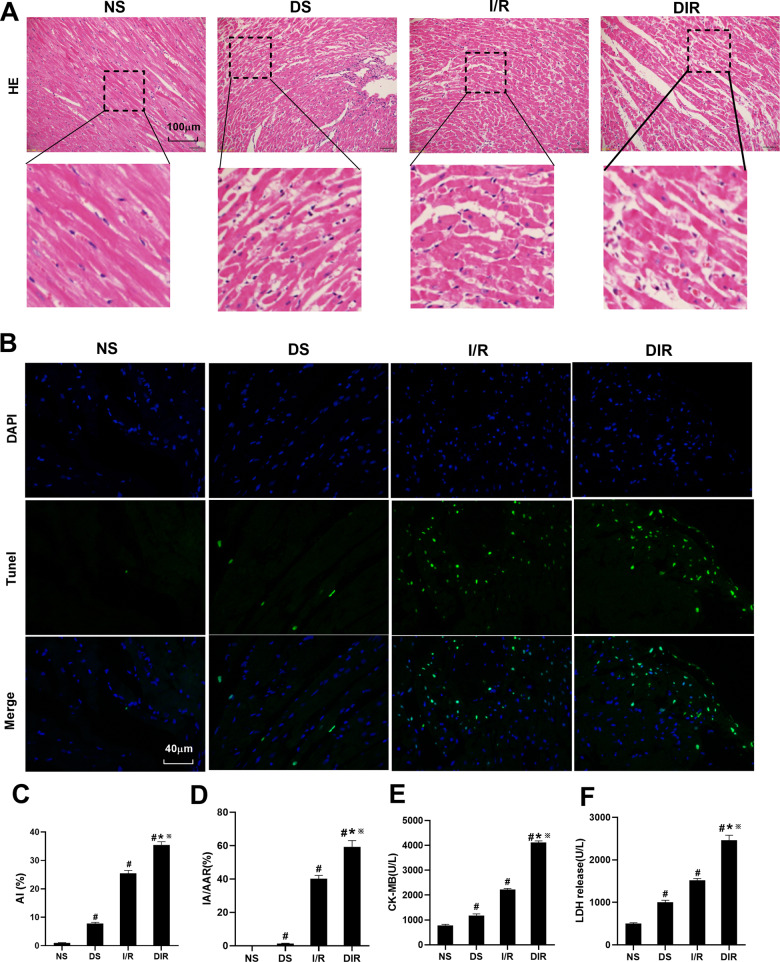
DS, I/R, and DIR rat models construction and verification. **A** Histopathological changes of myocardium were detected by HE staining. **B**, **C** The TUNEL staining for cell apoptosis rates were detected in different rat groups. **D** The myocardial infarction area was detected and expressed by AI/AAR (%). **E** CK-MB in serum was detected. **F** LDH in serum was detected. Results are presented by means ± SD. *n* = 8 per group. ^#^*P* < 0.05 compared with the NS group. *P < 0.05 compared with the DS group. ^※^*P* < 0.05 compared with the I/R group.

DS, I/R, and DIR models were first constructed, respectively, to detect the pathological changes of myocardial tissue, myocardial cell apoptosis rate, myocardial infarction area, serum CK-MB, and LDH levels in the three model rats, to determine whether the models were successfully constructed. As shown in Fig. 1A, the myocardial tissue structure in the NS group was dense and tidy, the myocardial fibers were intact, and no myocardial fibers were broken. The myocardial tissue in DS and I/R groups showed mild disorder of cell arrangement and a few myocardial fibers were broken. In the DIR group, the myocardial tissue showed severe cell alignment disorder, the cells were swollen, and most of the myocardial fibers were broken. As shown in Fig. 1B, TUNEL method was used to assess cell apoptosis in myocardial tissue. Under the fluorescence microscope, blue represents nuclear staining, and green represents stained apoptotic cardiomyocytes. The quantitative detection results in Fig. 1C show that compared with the NS group, the level of apoptosis rate, in DS, I/R, and DIR group was increased (*P* < 0.05). Compared with DS and I/R group, the level of apoptosis rate was increased in the DIR group (P < 0.05). As shown in Fig. 1D–F, compared with NS group, the level of IA/AAR, CK-MB, and LDH was increased in DS, I/R, and DIR group (*P* < 0.05). Compared with the DS and I/R group, the level of IA/AAR, CK-MB, and LDH was increased in the DIR group (*P* < 0.05). Left ventricular function was assessed by measuring hemodynamic parameters (Table 3). Compared with the NS group, HR, left ventricular systolic pressure (LVSP), +dp/dt, and −dp/dt were elevated in the DS group (*P* < 0.05). At the time of 10 min before ischemia, the hemodynamic parameters in the DIR group were lower than those in the corresponding I/R group (*P* < 0.05). At the time of 2 h after reperfusion, the hemodynamic parameters in the I/R and DIR groups were significantly lower than those in the corresponding baseline (*P* < 0.05), the hemodynamic parameters in the I/R and DIR groups were significantly decreased than that in NS group (*P* < 0.05), and the hemodynamic parameters in the DIR group were decreased than that in I/R group (*P* < 0.05). Moreover, the Supplemental Material 1 as a supplement to Fig. 1D specifically showed the infarcted area of myocardial tissue.

**Table 3:** Hemodynamic parameters at baseline after 2 h of reperfusion.

	HR (bmp)	LVSP (mm Hg)	+dp/dt (mm Hg/s)	dp/dt (mm Hg/s)
NS	351 ± 24	119 ± 10	6379 ± 575	4659 ± 391
DS	310 ± 16^a^	103 ± 10^a^	5309 ± 380^a^	3538 ± 341^a^
10 min Before ischemia (baseline)				
I/R	356 ± 21	122 ± 9	6423 ± 559	4721 ± 415
DIR	311 ± 14^b^	105 ± 11^b^	5320 ± 296^b^	3578 ± 339^b^
DIR + 5-aza-CdR	315 ± 18	106 ± 9	5354 ± 408	3587 ± 254
2 h After reperfusion				
I/R	299 ± 20^a,c^	98 ± 6^a,c^	4619 ± 369^a,c^	3179 ± 240^a,c^
DIR	226 ± 14^a–c^	81 ± 7^a–c^	2589 ± 213^a–c^	2004 ± 182^a–c^
DIR + 5-aza-CdR	244 ± 10^c,d^	90 ± 6^c,d^	3626 ± 305^c,d^	2798 ± 254^c,d^

Results are expressed as mean ± SD, *n* = 8.

*DIR* diabetes mellitus + I/R, *DIR* *+* *5-aza-CdR* diabetes mellitus + I/R + 5-aza-CdR, *DS* diabetes mellitus + sham, *HR* heart rate, *I/R* ischemia/reperfusion, *LVSP* left ventricular systolic pressure, *NS* normal + sham.

^a^*P* < 0.05 vs. NS group.

^b^*P* < 0.05 vs. their corresponding I/R group.

^c^*P* < 0.05 vs. their corresponding baseline.

^d^*P* < 0.05 vs. their corresponding DIR group.

### The level of DNMT-1 and ferritinophagy-ferroptosis axis in DS, I/R and DIR rats

**Fig. 2:**
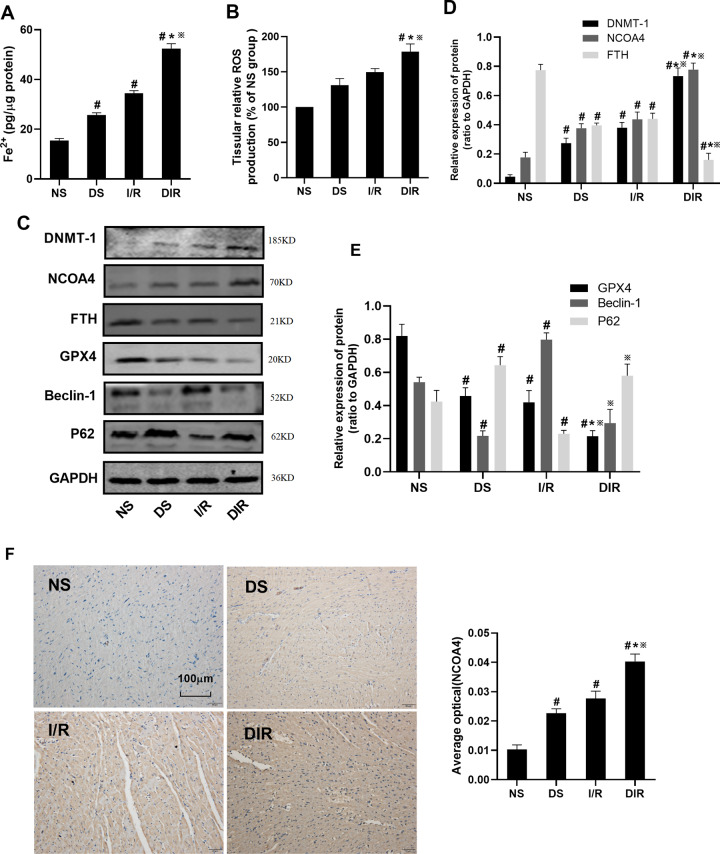
The level of DNMT-1 and ferritinophagy-ferroptosis axis in DS, I/R, and DIR rats. **A** Fe^2+^ in myocardial tissue was detected. **B** ROS in myocardial tissue was detected. **C**–**E** The protein expression of DNMT-1, NCOA4, FTH, GPX4, Beclin-1, and p62 in myocardial tissue was detected by western blotting. **F** The expression of NCOA4 in myocardial tissue was detected by IHC. Results are presented by means ± SD. *n* = 8 per group. ^#^*P* < 0.05 compared with the NS group. *P < 0.05 compared with the DS group. ^※^*P* < 0.05 compared with the I/R group.

Subsequently, we verified the levels of ferroptosis, ferritinophagy, and DNMT-1 in DS, I/R, and DIR rat models. As shown in Fig. 2A, B, compared with the NS group, the level of Fe^2+^ and ROS was increased in DS, I/R, and DIR group (*P* < 0.05). Compared with DS and I/R group, the level of Fe^2+^ and ROS was further increased in the DIR group (*P* < 0.05). As shown in Fig. 2C–E, compared with the NS group, the protein level of DNMT-1 and NCOA4 was increased and the level of FTH and GPX4 was decreased in the DS, I/R, and DIR group (*P* < 0.05). Compared with the DS and I/R group, the protein level of DNMT-1 and NCOA4 was further increased and the level of FTH and GPX4 was decreased in the DIR group (*P* < 0.05). However, for the autophagy proteins, the level of Beclin-1 was decreased in the DS group (*P* < 0.05) and increased in the I/R group (*P* < 0.05), when compared with the NS group. Moreover, compared with the DIR group, the Beclin-1 protein level in the I/R group was increased (*P* < 0.05) and it was decreased in the DS group, but it was not statistically significant. The trend of P62 protein level among the groups was opposite to the trend of Beclin-1 protein. Moreover, as shown in Fig. 2F, the results of immunohistochemical showed that the expression level of and NCOA4 was increased in DS, I/R, and DIR group, when compared with the NS group (*P* < 0.05). Compared with the DS and I/R group, the level of NCOA4 was further increased in the DIR group (*P* < 0.05).

### Effect of inhibition of DNMT-1 on left ventricular function, pathological changes, and ferritinophagy-ferroptosis axis in myocardial tissue

**Fig. 3:**
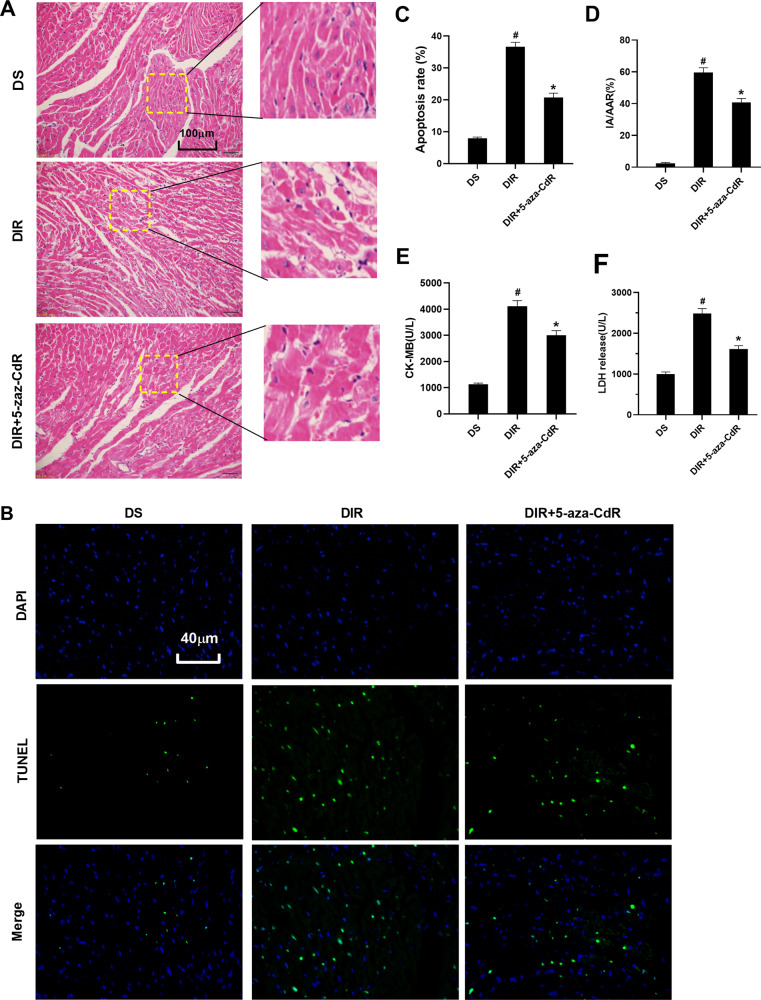
Effect of DNMT-1 on pathological changes and enzyme in myocardial tissue. **A** Histopathological changes of myocardium were detected by HE staining. **B** The TUNEL staining for cell apoptosis rates were detected in different rat groups. **C** Cell apoptosis was shown in each group by histogram. **D** The myocardial infarction area was detected and expressed by AI/AAR (%). **E** CK-MB in serum was detected. **F** LDH in serum was detected. Results are presented by means ± SD. *n* = 8 per group. ^#^*P* < 0.05 compared with the DS group. *P < 0.05 compared with the DIR group.

**Fig. 4:**
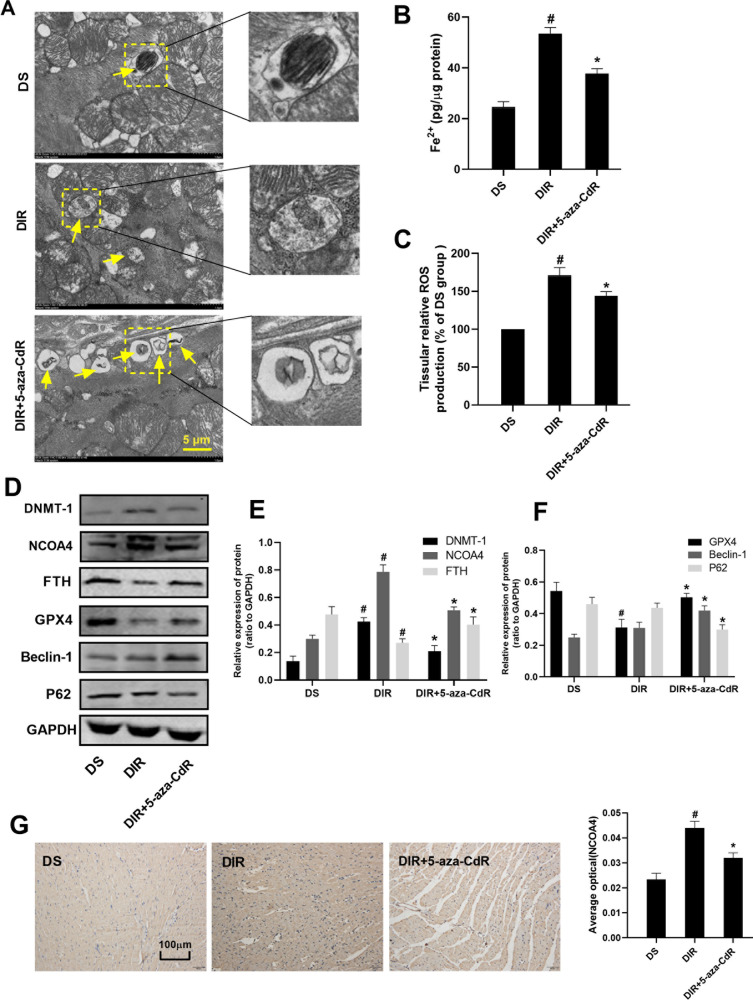
Effect of DNMT-1 on autophagy-ferroptosis axis in myocardial tissue. **A** The changes of autophagosome in myocardial tissue were detected by electron microscopy. **B** Fe^2+^ in myocardial tissue was detected. **C** ROS in myocardial tissue was detected. **D**–**F** The protein expression of DNMT-1, NCOA4, FTH, GPX4, Beclin-1, and p62 in myocardial tissue was detected by western blotting. **G** The expression of NCOA4 in myocardial tissue was detected by IHC. Results are presented by means ± SD. *n* = 8 per group. ^#^*P* < 0.05 compared with the DS group. *P < 0.05 compared with the DIR group.

For the left ventricular function shown in Table 3, at the time of 2 h after reperfusion, the hemodynamic parameters in the DIR + 5-aza-CdR groups were significantly lower than those in the corresponding baseline (*P* < 0.05). However, compared with the DIR group, 5-aza-CdR treatment elevated the levels of HR, LVSP, +dp/dt, and −dp/dt in the DIR + 5-aza-CdR group (*P* < 0.05), suggesting that 5-aza-CdR could improve cardiac function during myocardial I/R in diabetic rats. As shown in Fig. 3A, the degree of myocardial tissue lesions in the DIR + 5-aza-CdR group was significantly reduced compared with that in the DIR group. As shown in Fig. 3B–F, compared with the DIR group, the levels of apoptosis rate, IA/AAR, CK-MB, and LDH were decreased in the DIR + 5-aza-CdR group (*P* < 0.05). As shown in Fig. 4A and Supplemental Material 2A, transmission electron microscopy results showed that the number of autophagosomes in the myocardial tissue of the DS group was the least level. Compared with the DS group, the number of autophagosomes in the DIR group increased, but it was not statistically significant. After 5-aza-CdR intervention, the number of autophagosomes further increased. As shown in Fig. 4B, C, compared with the DIR group, the level of Fe^2+^ and ROS was decreased in the DIR + 5-aza-CdR group (*P* < 0.05). As shown in Fig. 4D–F, compared with the DS group, the protein level of DNMT-1 and NCOA4 was increased and the level of FTH and GPX4 was decreased in the DIR group (*P* < 0.05). The protein level of Beclin-1 was increased and the protein level of P62 was decreased, but this was not statistically significant when compared with the DS group. Compared with the DIR group, the protein level of DNMT-1, NCOA4, and P62 was decreased and the level of FTH, GPX4, and Beclin-1 was increased in the DIR group (*P* < 0.05). As shown in Fig. 4G, after being administered with 5-aza-CdR, the expression of NCOA4 was decreased, when compared with that in the DIR group (*P* < 0.05).

### Effect of inhibition of DNMT-1 on the ferritinophagy-ferroptosis axis of HH/R H9c2 cells

5-Aza-CdR is not a specific inhibitor for DNMT-1. To verify the effect of the inhibitor on damaged H9c2 cell cardiomyocytes, we compared the effect between siRNA of DNMT-1 and 5-aza-CdR. As shown in Supplemental Material 3A, B, the DNMT-1 protein level was detected to verify the transfection effects. Compared with normal group, the DNMT-1 protein level was decreased in the DNMT-1-siRNA group (*P* < 0.05). As shown in Supplemental Material 3C, D, compared with the HG group, the expression of DNMT-1 protein was increased in the HH/R group (*P* < 0.05). Compared with the HH/R group, the DNMT-1 protein level in HH/R + 5-Aza-CdR and HH/R + DNMT-1-siRNA group was decreased (*P* < 0.05). Compared with the HH/R + 5-Aza-CdR group, the level of DNMT-1 protein was slightly downregulated in the HH/R + DNMT-1-siRNA group, but the difference was not statistically significant (*P* > 0.05). As shown in Supplemental Material 3E–I, compared with the HG group, the cell viability and GSH in the supernatant in the HH/R group was decreased (*P* < 0.05). The levels of LDH in the supernatant, cellular ROS, and Fe^2+^ was increased (*P* < 0.05). After being intervened with 5-aza-CdR, the cell viability and GSH in the supernatant were increased (*P* < 0.05). The levels of LDH in the supernatant, cellular ROS and Fe^2+^ were decreased (*P* < 0.05). When compared with the HH/R + 5-Aza-CdR group, this was no significant statistical difference among the above indicators in the HH/R + DNMT-1-siRNA group (*P* > 0.05). It can be concluded from the above results that siRNA of DNMT-1 and 5-aza-CdR have the same protective effect on damaged H9c2.

**Fig. 5:**
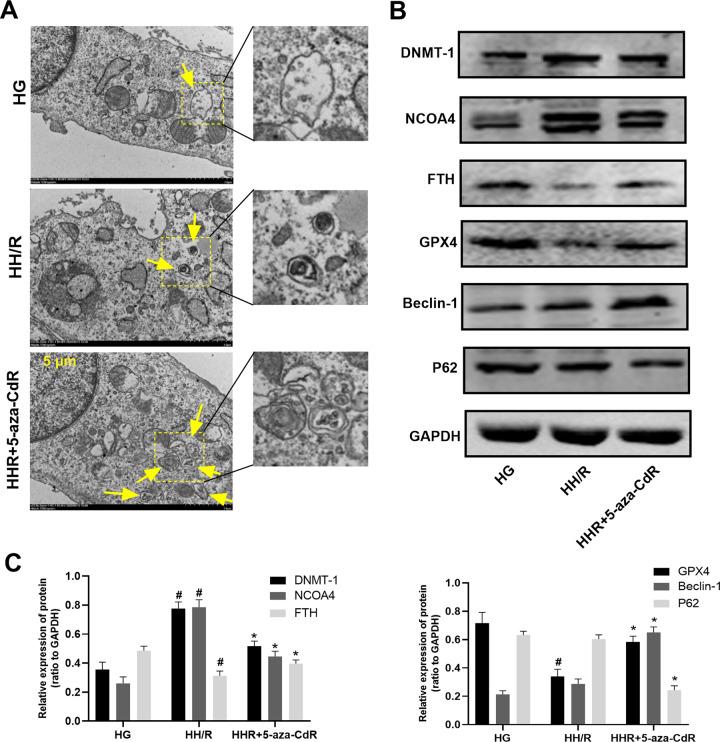
Effect of DNMT-1 on the autophagy-ferroptosis axis of HH/R H9c2 cells. **A** The changes of autophagosome in H9c2 cells were detected by electron microscopy. **B**, **C** The protein expression of DNMT-1, NCOA4, FTH, GPX4, Beclin-1, and p62 in H9c2 cells was detected by western blotting. Results are presented by means ± SD. *n* = 6 per group. ^#^*P* < 0.05 compared with the HG group. *P < 0.05 compared with the HH/R group.

**Fig. 6:**
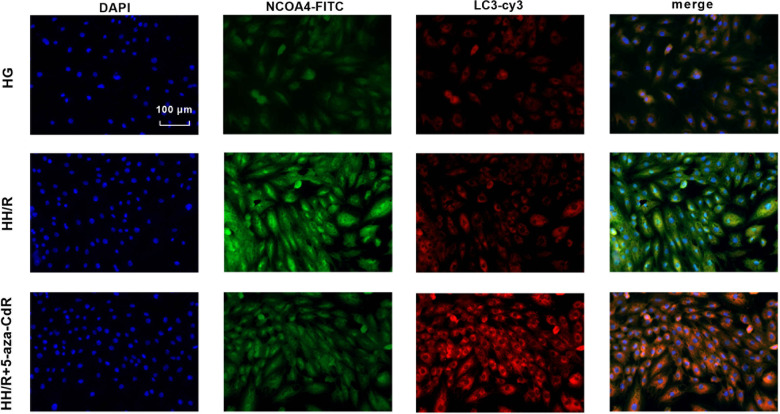
Ferritinophagy was detected by LC3 and NCOA4 colocation by immunofluorescence. Effect of DNMT-1 on ferritinophagy in HH/R H9c2 cells.

**Fig. 7:**
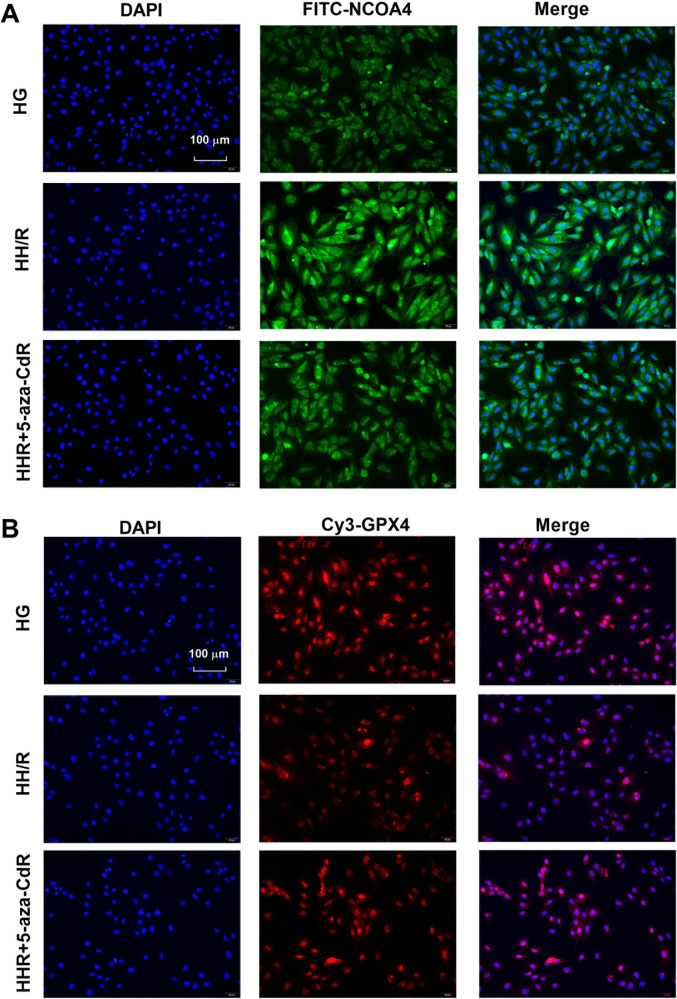
The expression of NCOA4 and GPX4 was detected by immunofluorescence. Effect of DNMT-1 on NCOA4 (**A**) and GPX4 in HH/R H9c2 cells (**B**).

As shown in Fig. 5A and Supplemental Material 2B, transmission electron microscopy results showed that the number of autophagosomes in the H9c2 cells of the HG group was the least. Compared with the HG group, the number of autophagosomes in the HH/R group increased, but it was not statistically significant. After 5-aza-CdR intervention, the number of autophagosomes further increased. As shown in Fig. 5B, C, compared with the HG group, the protein expression of GPX4 and FTH in the HH/R group was decreased (*P* < 0.05). The protein expression of NCOA4 and DNMT-1 was increased (*P* < 0.05). When compared with the HG group, the protein level of Beclin-1 was increased and the protein level of P62 was decreased, but this was not statistically significant. After being intervened with 5-aza-CdR, the protein expression of FTH, Beclin-1, and GPX4 was increased (*P* < 0.05). The protein expression of DNMT-1, NCOA4, and P62 was decreased (*P* < 0.05), when compared with the HH/R group. The immunofluorescence was further used to detect the expression of NCOA4 and GPX4 proteins. Moreover, ferritinophagy was detected by LC3 and NCOA4 colocation. As shown in Fig. 6, compared with the HG group, autophagy-related molecular LC3 in the HH/R group was increased slightly, but this was not statistically significanct. The level of NCOA4 was increased (*P* < 0.05). Compared with the HH/R group, the level of LC3 was increased in the HH/R + 5-aza-CdR group and the level of NCOA4 was decreased (*P* < 0.05). As shown in Fig. 7A, B, compared with the HG group, the expression of NCOA4 was increased and that of GPX4 was decreased in the HH/R group (*P* < 0.05). After being intervened with 5-aza-CdR, the expression of NCOA4 was decreased and that of GPX4 was increased (*P* < 0.05).

### Effect of inhibition of DNMT-1 on the ferritinophagy-ferroptosis axis of HH/R H9c2 cells through NCOA4

**Fig. 8:**
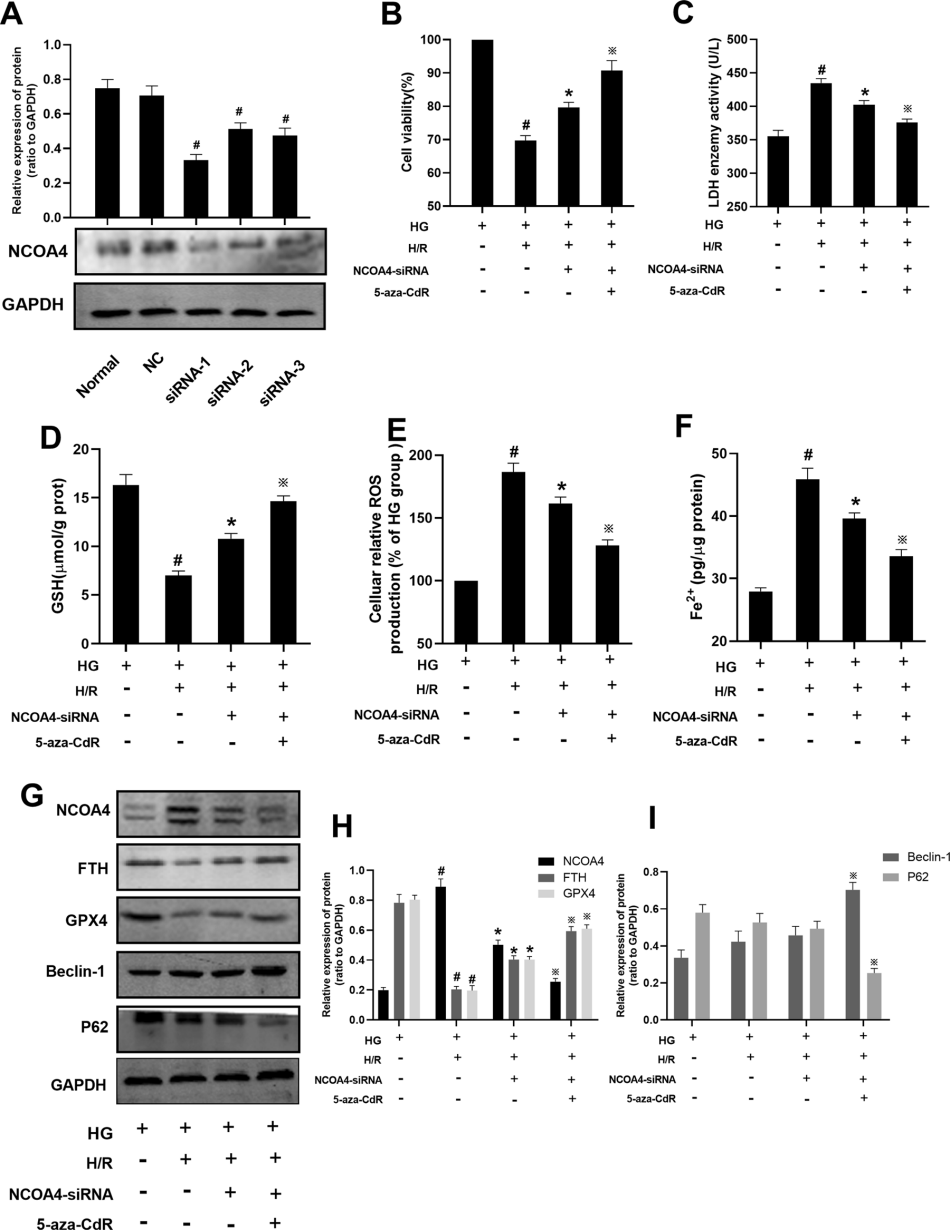
Effect of DNMT-1 on the autophagy-ferroptosis axis of HH/R H9c2 cells through NCOA4. **A** The protein expression of NCOA4 was detected by western blotting. **B** Cell viability was detected by CCK-8. **C**–**F** LDH, Fe^2+^, GSH, cellular ROS production was detected. The protein expression of NCOA4, FTH, GPX4, Beclin-1, and p62 in H9c2 cells was detected by western blotting. Results are presented by means ± SD. *n* = 6 per group. ^#^*P* < 0.05 compared with the HG group. *P < 0.05 compared with the HH/R group. ^※^*P* < 0.05 compared with the HH/R + NCOA4-siRNA group.

In order to test the effect of NCOA4 on HH/R cells, siRNA was used to knock down NCOA4 in H9c2 cells. As shown in Fig. 8A, there was no change for the NCOA4 protein level between the normal group and NC group, which indicated that the siRNA sequence itself did not affect the mRNA level of NCOA4. When compared with the normal group, the mRNA level of NCOA4 in siRNA-1, siRNA-2, and siRNA-3 groups were both decreased (*P* < 0.05). The least expression of NCOA4 protein level was in the siRNA-1 group (close to 50% of normal group). Therefore, siRNA-1 was used for the following intervention. After NCOA4 being knocked down for 48 h, the cells were processed with DNMT-1 for 24 h. Then, the NCOA4 cells were used to build HH/R cell model. As shown in Fig. 8B–I, compared with the HG group, the cell viability and GSH in the supernatant, the protein expression of GPX4, and FTH in the HH/R group was decreased (*P* < 0.05). The levels of LDH in the supernatant, cellular ROS and Fe^2+^, and the protein expression of NCOA4 was increased (*P* < 0.05). The protein level of Beclin-1 was increased and the protein level of P62 was decreased, but this was not statistically significant. Compared with the HH/R group, the cell viability and GSH in the supernatant, the protein expression of FTH, and GPX4 in HH/R + NCOA4-siRNA group was increased (*P* < 0.05). The levels of LDH in the supernatant, cellular ROS and Fe^2+^, the protein expression of NCOA4 was decreased (*P* < 0.05). The protein level of Beclin-1 was increased and the protein level of P62 was decreased, but this was no statistically significant. When compared with HH/R + NCOA4-siRNA group, the cell viability and GSH in the supernatant, the protein expression of FTH, GPX4 and Beclin-1 in HH/R + NCOA4-siRNA + 5-aza-CdR group was increased (*P* < 0.05). The levels of LDH in the supernatant, cellular ROS and Fe^2+^, and the protein expression of NCOA4 and P62 was decreased in the HH/R + NCOA4-siRNA-1 + 5-aza-CdR group (*P* < 0.05).

## Discussion

With the improvement of social living standards and lifestyle changes, diabetes, as an important systemic metabolic disease, has become one of the most challenging public health issues in the world today. In diabetic patients, because of hyperglycemia and end-glycosylation products, it can cause vascular endothelial dysfunction, fibrosis, inflammation, and oxidative stress. Diabetic cardiomyopathy can cause heart failure and death in 12% of diabetic patients [17]. Cardiovascular disease is one of the main causes of death of diabetic patients and more than half of diabetic patients suffer from cardiovascular disease [18]. Therefore, it is of great significance to explore the mechanism of diabetes-induced myocardial injury and to seek effective methods to prevent and treat myocardial injury.

Since 1963, de Duve and Wattiaux [19] has defined the degradation process (delivering cytoplasmic matter to lysosomes) as “autophagy” by high-resolution transmission electron microscopy. More and more studies have demonstrated that autophagy plays a key role in maintaining cell and tissue homeostasis by regulating various physiological processes, including pathogen clearance, antigen presentation, cytokine formation, inflammatory response, and innate and adaptive immune responses [20, 21]. Ferritinophagy, as a new type of autophagy, has a similar physiological effect. Ferritinophagy was first proposed by Mancias et al. [8]. The researchers identified NCOA4 as a receptor for selective autophagy through quantitative proteomics, which mediates the degradation of ferritin in autophagosomes. The ions bound to ferritin are released into free irons and this process is called ferritinophagy. Under normal physiological conditions, iron is stored in ferritin, and when there is a lack of iron in the cell, ferritin-containing ions combine with NCOA4 to form a complex that mediates ferritinophagy to release ions. NCOA4 can selectively recognize the FTH subunit in ferritin and FTH1 R23 has been proved to be an important and essential structure for the binding of ferritin to NCOA4 [22]. It was reported that after the NCOA4-ferritin complex was formed, it would bind to autophagy-related factor (ATG) 8 and then bind to the primary autophagosome. Then the primary autophagosome develops into an autophagosome, during which the autophagy-related genes Beclin-1 or p62 would be involved in the progress [8, 23]. NCOA4-mediated ferritinophagy could cause the degradation of ferritin and then promote the release of free iron, which is the key factor to cause ferroptosis.

Ferroptosis is a unique form of iron-dependent non-apoptotic regulatory cell death. It is formed by the destruction of GSH-dependent antioxidant defense mechanisms and the accumulation of lipid peroxides [24]. Ferroptosis does not cause changes in cell morphology, which is mainly manifested in the contraction of mitochondria and the increase of mitochondrial membrane density [24]. Synthetic lethal screening studies have identified several genes that cause ferroptosis and these genes may be involved in lipid and amino acid metabolism [25–27]. The chemical synthesis inhibitors of these genes can induce ferroptosis, such as GSH-dependent antioxidant enzyme-GPX4 inhibitor RAS-selective lethal small molecule 3, Na+-independent cystine/glutamate antiporter system xc- inhibitor sorafenib and Elastin [28–30]. Further research found that these ferroptosis inducers can cause ferritin storage protein and/or NCOA4 through the degradation of FTH, and to release the ferrous iron (Fe^2+^), these processes generate ROS through the fenton reaction, which in turn induces lipid peroxidation [31, 32]. The accumulation of lipid peroxidation and the consumption of plasma membrane polyunsaturated fatty acids are important causes of cell death [33, 34]. Ferritinophagy plays an important role in the regulation of ferroptosis, which is mainly through the regulation of iron balance in the cell. After ROS being produced, ferritinophagy is activated, which causes the degradation of intracellular ferritin, thereby increasing intracellular iron content. Intracellular iron overload causes the cells to accumulate a large amount of ROS in a short time and this is an important part of the occurrence of ferroptosis [35].

Epigenetics is the science of reversible changes in gene expression and function, and the generation of heritable phenotypes without changing the DNA sequence [36]. DNA methylation is one of the first and most important epigenetic modifications discovered [37]. DNA methylation plays an important role in the occurrence and development of type 2 diabetes (T2DM) and the persistent complications of T2DM [38]. The study found that the hyperglycemic environment can affect the level of DNA methylation. The promotion of DNA demethylation may be a self-repair mechanism of the body [39]. Therefore, it is worth investigating whether inhibiting the activity of DNMT-1 can alleviate DIR, which may provide a potential target for treatment.

In our study, we first constructed DS, I/R, and DIR models, respectively, to detect the pathological changes of myocardial tissue, myocardial cell apoptosis rate, myocardial infarction area, serum CK-MB, and LDH levels in the three model rats to verify the models were successfully constructed. DM was a risk factor in myocardial IRI. It is worth noting that, compared with the normal group, autophagy decreased in diabetes or HG state, but increased during IRI. Compared with the diabetes or HG group, diabetes IRI or H/HR group, autophagy increased slightly, but this was not statistically significant. This is because diabetes is a long-term pathological process and autophagy is inhibited. Although I/R is an acute pathological reaction and autophagy will exert a protective effect stressfully, it will increase. This is consistent with our previous report [40]. Different from autophagy, ferritinophagy and the release of iron ions and ferroptosis will be promoted during diabetes and IRI.

Subsequently, we verified that the levels of ferroptosis, ferritinophagy, and DNMT-1 in DS, I/R, and DIR rat models were increased. However, in the condition of diabetes, myocardium with I/R injury possesses a higher level of ferroptosis, ferritinophagy, and DNMT-1. Inhibition of DNMT-1 could not only improve the left ventricular function and pathological changes but also regulate the autophagy-ferroptosis axis in myocardial tissue. Therefore, in the next experiment, we directly perform hypoxia and reoxygenation treatment on H9c2 cells in a HG state to simulate the process of myocardial tissue IRI in vitro. Similarly, inhibition of DNMT-1 could alleviate the degree of cell injury and decrease the level of ferritinophagy and ferroptosis. Moreover, in order to test the effect of NCOA4 on HH/R cells, siRNA was used to knock down NCOA4 in the NCOA4-siRNA group. Further, 5-aza-CdR enhanced the protective effect for NCOA4-siRNA in the process of cell injury. In this study, intervention with NCOA4 did not cause changes in autophagy-related proteins Beclin-1 and P62, which may be because autophagy proteins and NCOA4 play parallel roles in the process of ferritinophagy.

In conclusion, inhibition of DNMT-1 could reduce ferroptosis during diabetes myocardial IRI and the NCOA4-mediated ferritinophagy may participate in the process. However, this study is just verified that inhibiting the activity of DNMT-1 generally promotes the expression of the molecule. As a key molecule that promotes ferritin degradation, NCOA4 plays a negative regulatory role in the ferroptosis process of cardiomyocytes. Inhibiting the expression of NCOA4 can alleviate cardiomyocyte damage. DNMT-1 inhibition by 5-aza-CdR could lead to alteration in many gene methylation to change their expression, not merely NCOA4. Whether DNMT-1 interact with NCOA4 promoter region should be further identified. This study provides an effective agent for the treatment of ischemic cardiomyopathy in diabetic conditions.

## Materials and methods

### Regents

H9c2 rat cardiomyocyte cell line was get from Wuhan Punosi Life Science and Technology Co., Ltd (Wuhan, China). DMEM low-sugar medium and FBS was purchased from HyClone (Logan, UT). The CCK-8 was purchased from Dojindo (Kumamoto, Japan). LDH, CK-MB, ROS, and GSH test kits were purchased from Nanjing Jiancheng Bioengineering Institute (Nanjing, China). Iron assay kit and GPX4 primary antibodies were purchased from Abcam (Cambridge, USA). Rabbit anti-rat primary antibodies DNMT-1 (catalog number 24206–1-AP), GPX4 (catalog number 67763-1-Ig), P62 (catalog number 18420-1-AP), Beclin-1 (catalog number 11306-1-AP), GAPDH (catalog number 60004-1-Ig), and Cy3- (catalog number SA00009-2) and FITC- (catalog number SA00003-2)-labeled goat anti-rabbit secondary antibodies were obtained from ProteinTech (Wuhan, China). Rabbit anti-rat primary antibodies NCOA4 (catalog number A5695) and FTH (catalog number A19544) were obtained from ABclonal (Wuhan, China). Second antibodies were purchased from LI-COR Biosciences (IRDye 800CW; catalog number 926-32219, LI-COR Corporate, USA). DNMT-1 inhibitor 5-aza-CdR were purchased from Selleck (Houston, USA). Lipofectamine 2000 was obtained from Invitrogen (San Diego, USA).

## Supplementary information

Supplemental materials

## Data Availability

The data used to support the findings of this study are available from the corresponding author upon request.
