# Supplementary material for: Inhibition of DNMT-1 alleviates ferroptosis through NCOA4 mediated ferritinophagy during diabetes myocardial ischemia/reperfusion injury
Source: Cell Death Discov. 2021 Sep 29;7:267. doi: 10.1038/s41420-021-00656-0 (PMC8481302; doi:10.1038/s41420-021-00656-0)

**Supplemental material 1**

The infarcted area of myocardial tissue (measuring of infarction/AAR) was detected by triphenyltetrazolium chloride determination. Compared with NS group, the level of IA/AAR was increased in DS, I/R, and DIR group. Compared with DS and I/R group, the level of IA/AAR, was increased in DIR group.

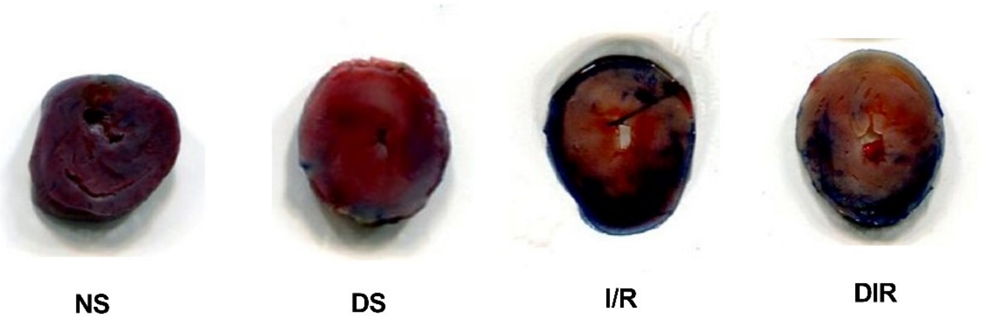

**Supplemental material 2**

The number of autophagosomes in H2c2 cells (A) and myocardial tissue (B) was shown by histogram**.**

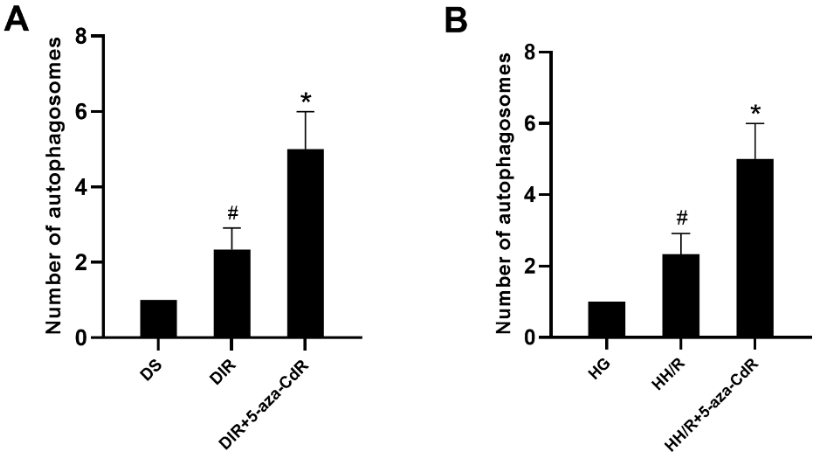

**Supplemental material 3**

Effect of inhibition of DNMT-1 on the ferritinophagy-ferroptosis axis of HH/R H9c2 cells. (A-C) The DNMT-1 protein and mRNA level were detected to verify the transfection effects. Compared with normal group, the DNMT-1 protein and mRNA levels were decreased in DNMT-1-siRNA group ($P < 0.05$). (D-E) Compared with HG group, the expression of DNMT-1 protein was increased in HH/R group ($P < 0.05$). Compared with HH/R group, the DNMT-1 protein level in HH/R+5-Aza-CdR and HH/R+DNMT-1-siRNA group was decreased ($P < 0.05$). Compared with HH/R +5-Aza-CdR group, the level of DNMT-1 protein was slightly down-regulated in HH/R+DNMT-1-siRNA group, but the difference was not statistically significant (P > 0.05). (F-J) Compared with HG group, the cell viability and GSH in the supernatant in HH/R group was decreased ($P < 0.05$). The levels of LDH in the supernatant, cellular ROS and $Fe^{2+}$ was increased ($P < 0.05$). After being intervened with 5-aza-CdR, the cell viability and GSH in the supernatant were increased ($P < 0.05$). The levels of LDH in the supernatant, cellular ROS and $Fe^{2+}$ were decreased ($P < 0.05$). When compared with HH/R +5-Aza-CdR group, this was no significant statistical difference among the above indicators in HH/R+DNMT-1-siRNA group ($P > 0.05$). It can be concluded from the above results that siRNA of DNMT-1 and 5-Aza-CdR have the same protective effect on damaged H9c2. Results are presented by means ± S.D. n=6 per group. #P < 0.05 compared with the HG group. *P < 0.05 compared with the HH/R group

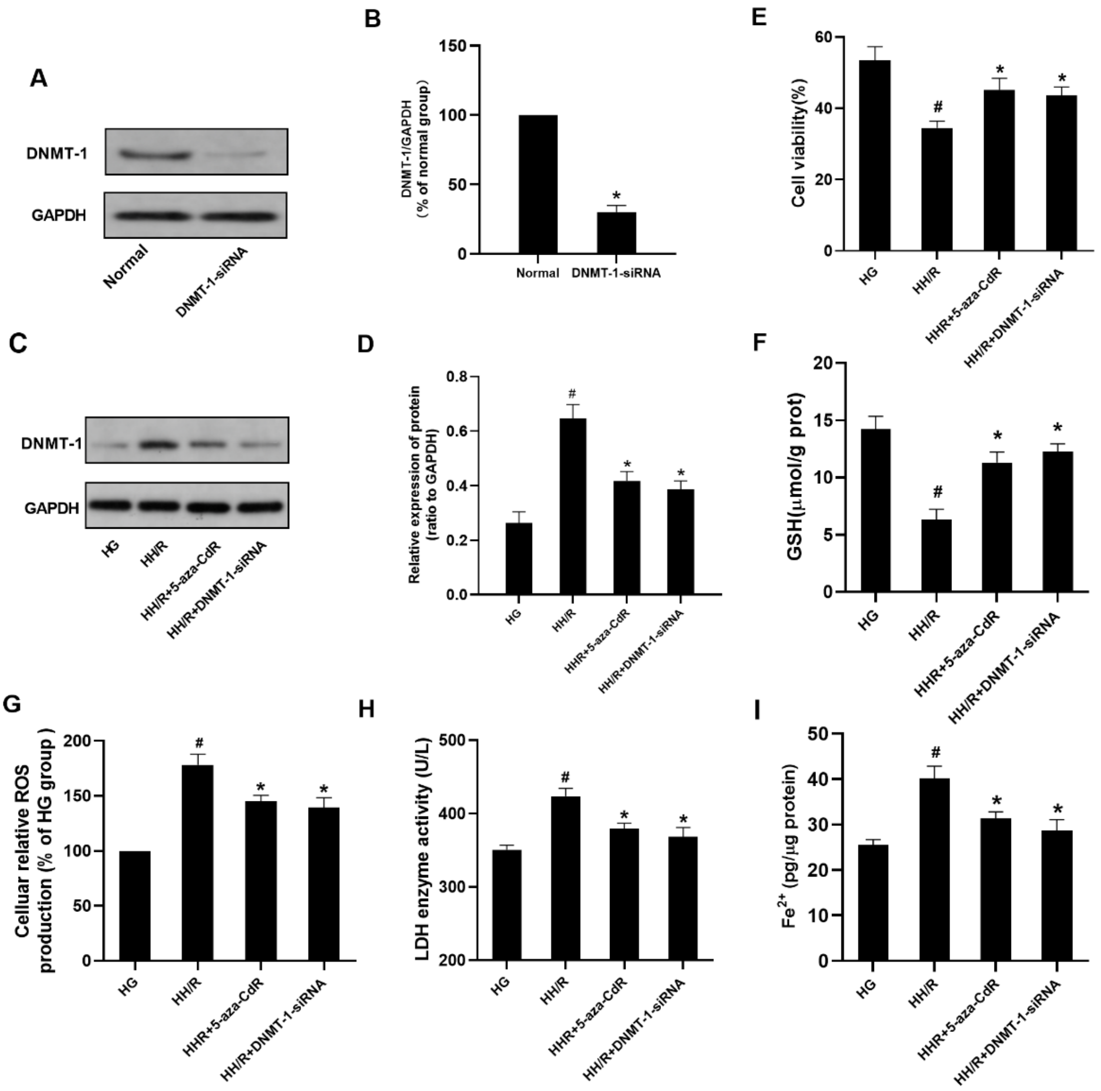
A
DNMT-1
GAPDH
Normal
DNMT-1-siRNA
B
DNMT-1/GAPDH (% of normal group)
150
100
50
0
*
Normal
DNMT-1-siRNA
E
Cell viability(%)
60
40
20
0
#
*
*
HG
HH/R
HHR+5-aza-CdR
HH/R+DNMT-1-siRNA
C
DNMT-1
GAPDH
HG
HH/R
HH/R+5-aza-CdR
HH/R+DNMT-1-siRNA
D
Relative expression of protein (ratio to GAPDH)
0.8
0.6
0.4
0.2
0.0
#
*
*
HG
HH/R
HHR+5-aza-CdR
HH/R+DNMT-1-siRNA
F
GSH(μmol/g prot)
20
15
10
5
0
#
*
*
HG
HH/R
HHR+5-aza-CdR
HH/R+DNMT-1-siRNA
G
Cellular relative ROS production (% of HG group)
200
150
100
50
0
#
*
*
HG
HH/R
HHR+5-aza-CdR
HH/R+DNMT-1-siRNA
H
LDH enzyme activity (U/L)
500
400
300
200
#
*
*
HG
HH/R
HHR+5-aza-CdR
HH/R+DNMT-1-siRNA
I
$Fe^{2+}$ (pg/μg protein)
50
40
30
20
10
0
#
*
*
HG
HH/R
HHR+5-aza-CdR
HH/R+DNMT-1-siRNA

Figure 2

DNMT-1

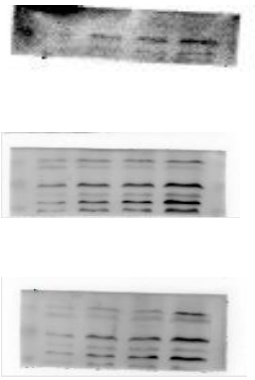

NCOA4

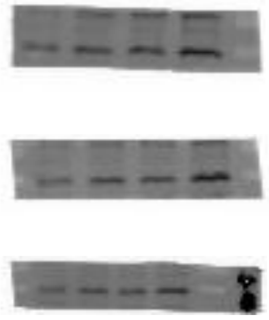

FTH

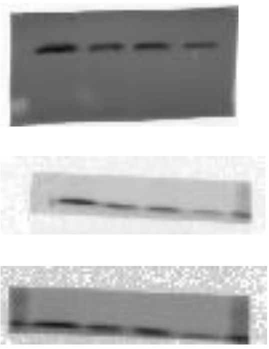

GPX4

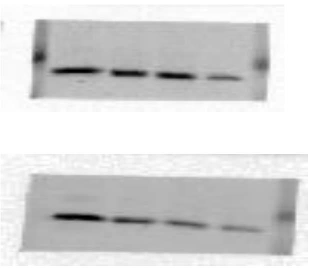

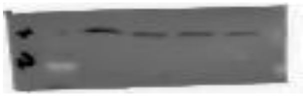

Beclin-1

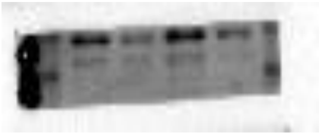

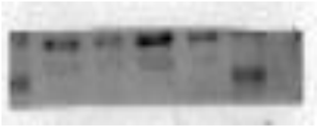

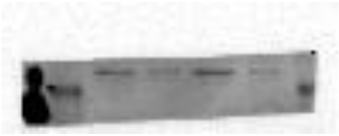

P62

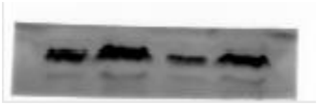

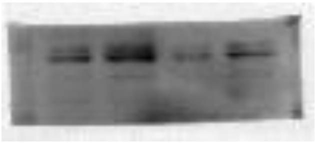

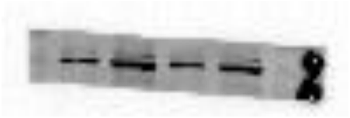

GAPDH

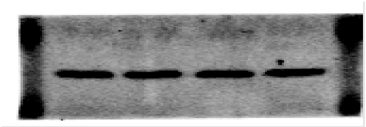

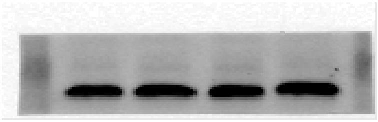

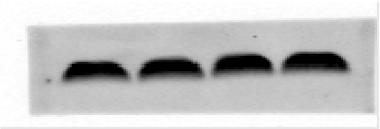

Figure 4

DNMT-1

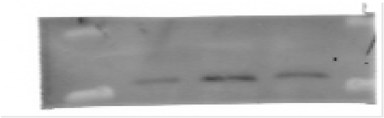

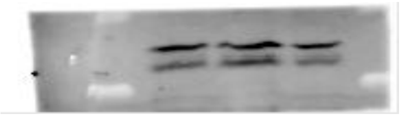

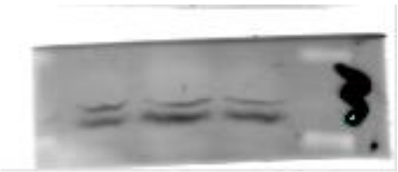

NCOA4

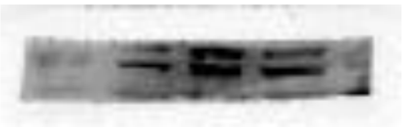

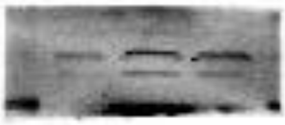

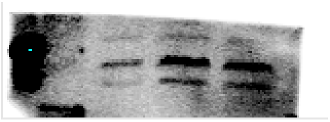

FTH

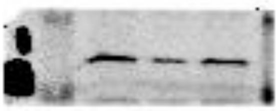

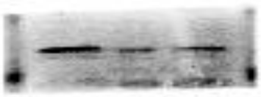

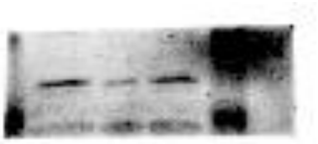

GPX4

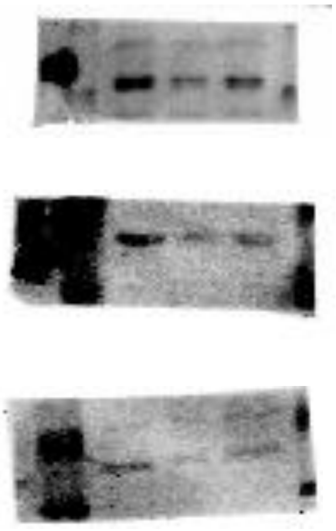

Beclin-1

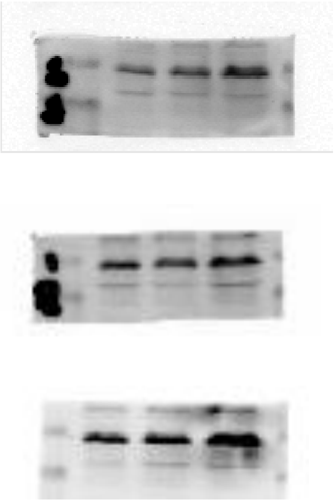

P62

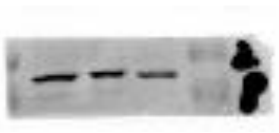

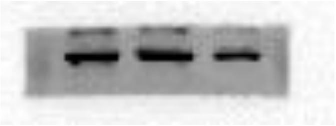

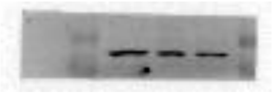

GAPDH

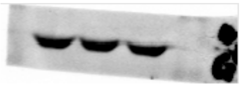

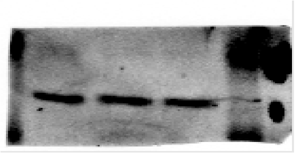

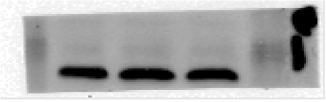

Figure 5

DNMT-1

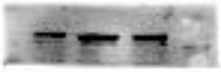

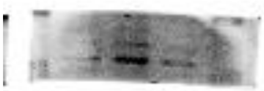

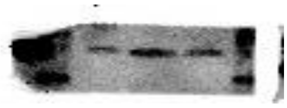

NCOA4

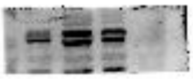

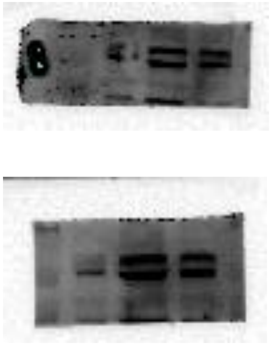

FTH

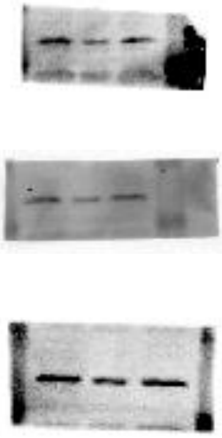

GPX4

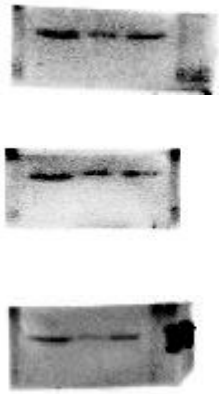

Beclin-1

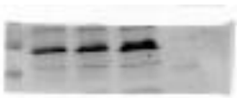

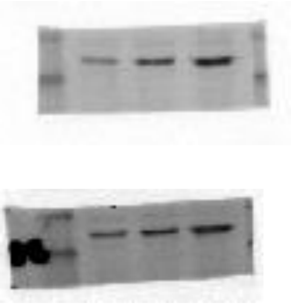

P62

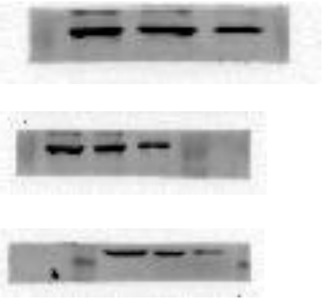

GAPDH

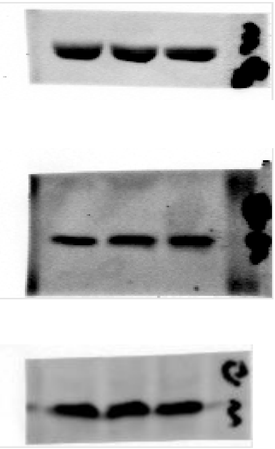

Figure 8A

NCOA4

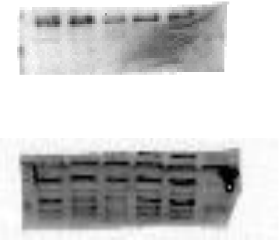

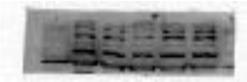

GAPDH

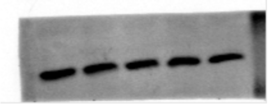

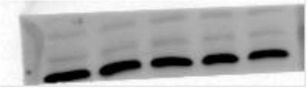

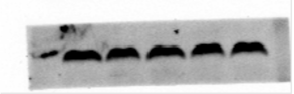

Figure 8G

NCOA4

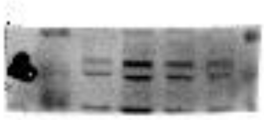

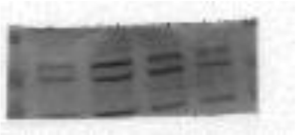

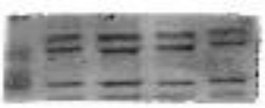

FTH

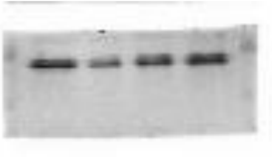

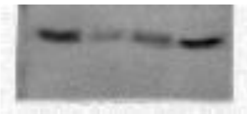

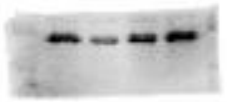

GPX4

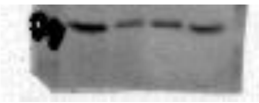

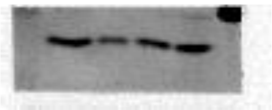

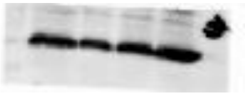

Bclin-1

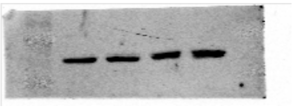

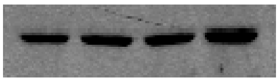

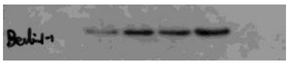

P62

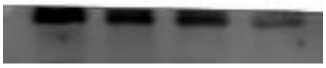

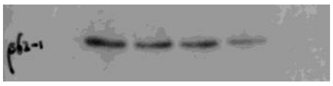

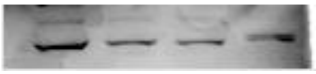

GAPDH

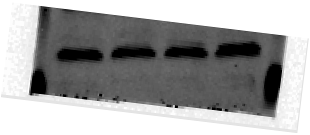

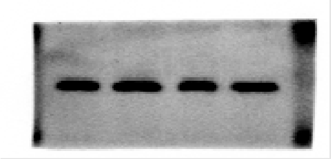

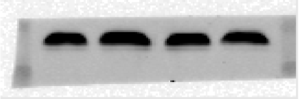

Supplemental materials 3A

DNMT-1

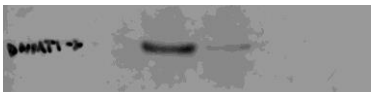

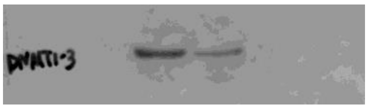

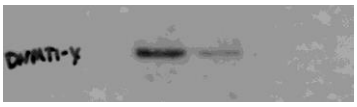

GAPDH

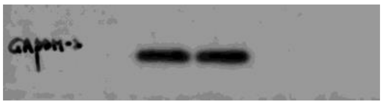

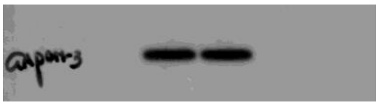

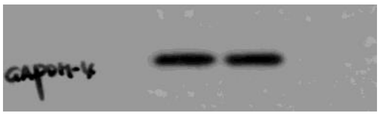

Supplemental materials 3C

DNMT-1

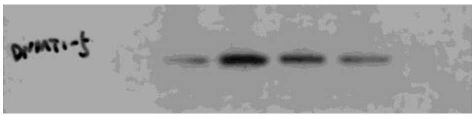

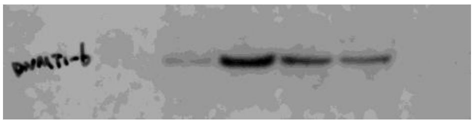


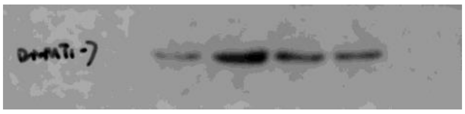


GAPDH

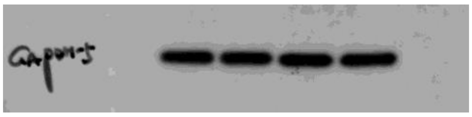


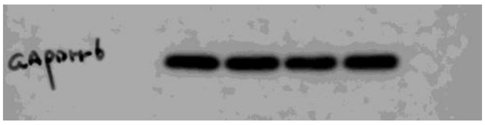


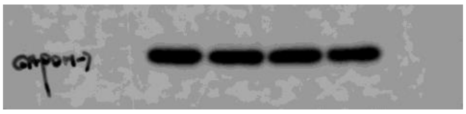

Supplement: Supplementary file 1 — Supplemental materials [file 41420_2021_656_MOESM1_ESM.pdf]
